# Oral Tegafur/folinic acid chemotherapy decreases phenytoin efficacy

**DOI:** 10.1038/sj.bjc.6601607

**Published:** 2004-02-03

**Authors:** N M L Veldhorst-Janssen, H H Boersma, M C T F M de Krom, R E N van Rijswijk

**Affiliations:** 1 Department of Clinical Pharmacy & Toxicology, University Hospital Maastricht, Maastricht, The Netherlands; 2 Department of Neurology, University Hospital Maastricht, Maastricht, The Netherlands; 3 Department of Internal Medicine, University Hospital Maastricht, Maastricht, The Netherlands

**Sir**,

We read with attention the paper of Brickell *et al* (*Br J Cancer* 2003; **89**(4):615–616). However, the contrary may also occur when an orally 5-FU derivative is combined with folinic acid. Recently, we observed an interesting case on decreased phenytoin levels associated with concurrent oral tegafur/uracil/calcium folinate therapy. A 53-year-old Caucasian male with a history of rectal carcinoma presented with several partial epileptic seizures with or without secondary generalisation due to brain metastasis. He received palliative chemotherapy consisting of orally administered tegafur/uracil/calcium folinate. To control the epilepsy, he was prescribed phenytoin. The patient developed inefficacy of the antiepileptic treatment due to the drug interaction of folinic acid from his chemotherapy regimen and phenytoin. The metabolic interaction between folinic or folic acid and phenytoin causing a decrease in phenytoin plasma levels is a well-known pharmacokinetic interaction (Lewis *et al*, 1995). It has been described that this interaction already takes place after daily intake of 15 mg folic acid during 2 weeks (Berg *et al*, 1983). In other therapy regimens of 5-FU and folinic acid, the administration is not longer than 5 days. This short period might be not long enough to result in decreased plasma levels of phenytoin.

The interaction between phenytoin and folinic acid is common, but less recognised in new treatment regimens, as shown in this case. Based on the unpredictable effects of folinic acid with or without 5-FU on phenytoin, we would like to advocate the use of antiepileptic drugs without interaction with chemotherapeutics, like gabapentin or levetiracetam, in patients with cerebral metastasis suffering epileptic seizures.
